# Multidimensional biological characteristics of ground glass nodules

**DOI:** 10.3389/fonc.2024.1380527

**Published:** 2024-05-22

**Authors:** Furong Chen, Jiangtao Li, Lei Li, Lunbing Tong, Gang Wang, Xuelin Zou

**Affiliations:** ^1^ Department of Oncology, The First People’s Hospital of Shuangliu District/West China (Airport) Hospital, Sichuan University, Chengdu, China; ^2^ Department of Respiratory and Critical Care Medicine, Clinical Research Center for Respiratory Disease, West China Hospital, Sichuan University, Chengdu, China; ^3^ Department of State Key Laboratory of Respiratory Health and Multimobidity, West China Hospital, Sichuan University, Chengdu, China; ^4^ Department of Respiratory Medicine, Chengdu Seventh People’s Hospital/Affiliated Cancer Hospital of Chengdu Medical College, Chengdu, China

**Keywords:** ground glass nodules, lung adenocarcinoma, pathology, genomic mutation, immune cell infiltration, immune microenvironment

## Abstract

The detection rate of ground glass nodules (GGNs) has increased in recent years because of their malignant potential but relatively indolent biological behavior; thus, correct GGN recognition and management has become a research focus. Many scholars have explored the underlying mechanism of the indolent progression of GGNs from several perspectives, such as pathological type, genomic mutational characteristics, and immune microenvironment. GGNs have different major mutated genes at different stages of development; *EGFR* mutation is the most common mutation in GGNs, and *p53* mutation is the most abundant mutation in the invasive stage of GGNs. Pure GGNs have fewer genomic alterations and a simpler genomic profile and exhibit a gradually evolving genomic mutation profile as the pathology progresses. Compared to advanced lung adenocarcinoma, GGN lung adenocarcinoma has a higher immune cell percentage, is under immune surveillance, and has less immune escape. However, as the pathological progression and solid component increase, negative immune regulation and immune escape increase gradually, and a suppressive immune environment is established gradually. Currently, regular computer tomography monitoring and surgery are the main treatment strategies for persistent GGNs. Stereotactic body radiotherapy and radiofrequency ablation are two local therapeutic alternatives, and systemic therapy has been progressively studied for lung cancer with GGNs. In the present review, we discuss the characterization of the multidimensional molecular evolution of GGNs that could facilitate more precise differentiation of such highly heterogeneous lesions, laying a foundation for the development of more effective individualized treatment plans.

## 1 Introduction

Due to the greater use of high-resolution cross sectional imaging, an increasing number of cases with pulmonary nodules have been detected each year (1, 2). Pulmonary nodules can be classified into solid nodules (SNs), part-solid nodules (PSNs), which consist of a portion of SN and ground-glass opacity (GGO) components, or pure ground glass nodules (GGNs), according to radiographic textures. GGNs are defined as pulmonary parenchymal blurred opacity observed on CT scans that do not obscure the underlying bronchi and pulmonary vascular structures (3). They have malignant potential but relatively indolent biological behavior; therefore, their proper recognition and management have become a research focus. Administrative strategies for GGNs are mainly generated based on their radiological characteristics, such as the maximum diameter of the nodule, proportion of solid components, and growth rate (4). However, no consensus has been reached for surgical intervention timing and computed tomography (CT) monitoring frequency during conservative treatment (5–10). The clinical practice also frequently encounters cases that contradict existing guidelines (11). Different pathologic types can manifest as GGNs; in particular, minimally invasive adenocarcinoma (MIA) or invasive adenocarcinoma (IAC)-presenting GGNs are more likely to proliferate aggressively and have been the subject of numerous clinical investigations (12–17). Currently, there are not many clinical studies on GGNs alone. We reviewed the prior clinical studies on GGNs alone or including GGNs and outlined the underlying molecular characteristics and immunological features of the indolent biological behavior of GGNs to provide a theoretical foundation for more individualized clinical decision-making and management.

## 2 Pathological features of GGNs

Pathologically, GGNs may be benign lesions (including focal interstitial fibrosis, inflammation, and hemorrhage) or malignant tumors (precancerous lesions and invasive lung adenocarcinoma [ADC]) (18).

GGNs can also be caused by inflammation, and infectious diseases are the main factors, such as mycoplasma, cytomegalovirus, and pneumocystis jirovecii pneumonia (19–21). GGNs caused by mycoplasma infection are characterized by lobular distribution, which may appear as solid micronodules or consolidations as the disease progresses (19). While GGO with cytomegalovirus could manifest as dense consolidation, bronchial wall thickening, or bronchiectasis (20), CT findings of *P. jirovecii* infection may present as an isolated ground glass infiltrate without other radiographic changes (21). One less common cause of GGO could be pulmonary hemorrhage, with varying etiology. In the condition, lesions can always be observed on CT scans, such as GGO and interlobular septal thickening, in addition to a ground-glass halo surrounding the pulmonary hemorrhagic foci and a patchy shadow moving down the hilum toward the periphery of pulmonary hemorrhage.

Atypical adenomatous hyperplasia (AAH) and adenocarcinoma *in situ* (AIS) are precancerous lesions that manifest as GGNs. AAH is defined as the focal hyperplasia of atypical cells located within the alveolar wall and respiratory bronchioles with irregular borders, consisting of alveolar type II cells and/or Clara cells (22). AAH appears as low-density pure GGO on CT images, usually less than 5 mm in size; however, some can reach 12 mm (18). AIS refers to the complete growth of ADC cells along the alveolar wall (adherent-like) without stromal, vascular, or pleural invasion, with no tumor cells in the alveolar space. On CT images, non-mucinous AIS usually presents as pure GGO, whereas mucinous AIS presents as SN or PSN (23).

MIA and IAC can also present as GGNs. MIA is observed as small solitary ADCs with tumor penetration of no more than 5 mm into the stroma and no pleural, lymphatic, or blood vessel invasions. MIA is usually non-mucinous but may be mucinous in rare cases. Non-mucinous MIA appears as a PSN in the CT scan, with the solid component being less than 5 mm (24). Mucinous MIA can be classified as SN or PSN (25). The histological subtypes of IAC are defined as a tumor invasion greater than 5 mm or invasion into the lymphatic vessels, vessels, and pleura. They can be divided into squamous, acinar, micropapillary, papillary, and solid invasive ADCs according to their histological subtypes. On CT scans, they may present as PSNs containing varying amounts of solid and ground glass. In the future, with the continuous development of artificial intelligence, imaging histology can improve the recognition of benign and malignant GGNs, thus providing more appropriate diagnosis and treatment options for patients.

## 3 Gene mutations in GGNs

### 3.1 Mutational characteristics in different phases

AAH, AIS, MIA, and IAC are currently diagnosed based on morphological analysis. However, this method does not accurately capture the biological characteristics of GGNs. In addition, although multiple studies have investigated mutational alterations in lung cancer (26), the gene mutational characteristics for the initiation and early progression of precancerous lesions remain poorly understood. Recent findings on gene mutation signatures in ground glass nodules have been summarized in **Table 1**.

**Table 1:** Results of studies reporting gene mutation signatures in ground glass nodules.

Author	Publication year	Country	Method	Number of cases	Number of nodules	Image findings	Pathological type	Main conclusions
Park et al.	2018 (27)	South Korea	NGS	16	52	Unknown	AAH/20, AIS/9, MIA/7, ADC/16	ADC and synchronous GG/L nodules are independently inherited tumors. AAH showed high mutation rates of *KRAS*, *BRAF*, and *EGFR*; ADC showed the highest incidence of *EGFR* mutations.
Ren Y et al.	2019 (28)	China	TRS, mWES	31	69	SM-GGN/69	Normal lung tissue/18, AAH/25, AIS/21, MIA/10, ADC/12	The most common mutations in GGN are in *BRAF*, *EGFR*, and *KRAS*. Precancerous unstable CNVs with a potentially predisposing genetic background may promote driver mutations and independent SM-GGN.
Hu X et al.	2019 (29)	China, Japan	mWES	53	116	IPN/116	AAH/22, AIS/27, MIA/54, IAC/13	APOBEC-mediated mutational processes play an important role during lung tumor formation and progression. The selective elimination of inappropriate subclones is responsible for neoplastic transformation of premalignant lesions.
Zhang et al.	2019 (12)	China	mWES	30	30	pure GGN/15, mixed GGN/8, SN/7	AIS/8, MIA/8, IAC/1	Mutations in *EGFR*, *ERBB2*, *NRAS*, and *BRAF* are early cloned genomic events in lung adenocarcinoma development, while *TP53* mutations, cell migration, gap junctions, and metastases are late events. Immune editing and genomic intratumoral heterogeneity are common early phenomena.
Chen et al.	2019 (13)	China	WES, RNA−Seq	197	197	Unknown	AIS/24, MIA/74, IAC/99	*EGFR*, *RBM10*, *BRAF*, *ERBB2*, and other genes are frequently mutated in precancerous lesions, and *TP53* is key for lung cancer invasion. TMB, *TP53* mutations, leukocyte antigen loss of heterozygosity, and arm level copy number variations all progressively increase during tumor progression.
Li et al.	2020 (30)	China	mWES	120	154	pure GGN/66, mixed GGN/87, NA/1	AAH/14, AIS/19, MIA/30, IAC/91	*EGFR* was the most frequently mutated in GGNs, followed by mutations in *RBM10*, *TP53*, *STK11*, and *KRAS*. *EGFR, TP53, RBM10*, and *ARID1B* mutation frequencies increased with SSNs with a solid component. Despite the predominance of multicentric origin, early metastatic events were detected in multifocal SSNs.
Cao et al.	2021 (14)	China	WES	37	79	GGN/79	MIA/58, AIS/15, IAC/6	Mutations in *EGFR, BRAF*, and *ERBB2* were more frequent in GGO, but mutations in driver genes *KRAS*, *MAP2K1*, and *NF1* were less frequent. Compared with IAC, GGO has relatively few genomic changes and a simpler genomic map.
Wu et al.	2021 (31)	China	WES	28	30	Pure GGN/8, mixed GGN/22	ADC/30	*EGFR* and *RBM10* were the most frequently mutated genes in GGN; *RBM10* may drive different lung cancer subtypes with better prognosis. GGN gene sets exhibit a high degree of intratumoral heterogeneity. In CT images, adherent growth predominant or pure GGN demonstrated a relatively low mutation burden.
Lim et al.	2021 (32)	South Korea	RNA-seq	9	36	Unknown	AAH/18, ADC/9, Normal lung tissue/9	*ACSL5* and *SERINC2* expression gradually increased as tissues changed from normal to AAH and ADC.

GGN, ground-glass nodule; SN, solid nodule; IPN, indeterminate pulmonary nodule; AAH, atypical adenomatous hyperplasia; AIS, adenocarcinoma in situ; MIA, minimally invasive adenocarcinoma; LPA, lepidic-predominant adenocarcinoma; IAC, invasive adenocarcinoma; mWES, multi-region whole-exome sequencing; WES, whole-exome sequencing; RNA-seq, transcriptome sequencing; TRS, targeted sequencing; NGS, next-generation sequencing; APOBEC, apolipoprotein B mRNA; GG/L, ground-glass/lepidic; CNV, Copy number variation; SM-GGN, multiple ground-glass nodule; SSNs, subsolid nodules.

In the preinvasive phase, *EGFR* is the most common gene mutation in GGNs, followed by *ERBB2*, *RBM10*, and *KRAS* mutations (12–14, 27, 28, 30, 31, 33–35). The exon 21 *L858R* mutation is reportedly the most common *EGFR* mutation among GGNs, followed by exon 19 deletion mutations (30, 33). GGNs with mutations in *RBM10* tend to present with a pathologically lepidic pattern. Lepidic pattern growth is characterized by tumor cell proliferation along intact alveolar walls. In addition, patients with lepidic pattern have a better prognosis than those with non-lepidic pattern tumors, suggesting that *RBM10* mutations are associated with a better prognosis (13, 31). However, in some studies on lung adenocarcinoma, *RBM10* acts as a tumor suppressor. When cancer is about to develop, *RBM10* is mutated to reduce its inhibitory effect on c-Myc, which ultimately promotes tumor growth (36, 37). More studies may be needed to determine the role of *RBM10* in the development of lung cancer. Affyl CoA synthase long chain (ACSL) plays a crucial role in the uncontrolled proliferation of cancer cells through abnormal lipid synthesis and extracellular lipid uptake (38). As the tissues change from normal to AAH and ADC, ACSL5 expression increases gradually, supporting the notion that ACSL5 contributes to tumor progression (32). In addition, *FUT2* and *SERINC2*, which are involved in metabolism, are aberrantly expressed in precancerous lesions, aiding the identification of early-stage lung cancer (32).

*EGFR*, *ERBB2*, *NRAS*, and *BRAF* mutations contribute to the early clonal genomic events in lung ADC. In contrast, mutations in *TP53* and genes associated with cell migration, gap junctions, and metastasis (such as *FRG1, DOCK7, SH3BP1*, and *GJA1*) may be late events that occur during subclonal diversification and tumor progression (12, 14). Similar proportions of *EGFR* mutations were observed in IAC and MIA/AIS, whereas *TP53* was mutated in IAC at a much higher proportion than in MIA/AIS (12). *TP53* mutation was the most abundant in the invasive phase, followed by *EGFR* and *RB1* mutations when compared with the preinvasive and microinvasive phases. The frequency of loss of heterozygosity of human leukocyte antigen (HLA) also increased in the invasive phase (13). Conversely, the mutation frequencies of *EGFR*, *RBM10*, and *TP53* in IAC cases presenting with GGNs were all significantly higher than those of AAH, AIS, and MIA (30).

**Figure 1:**
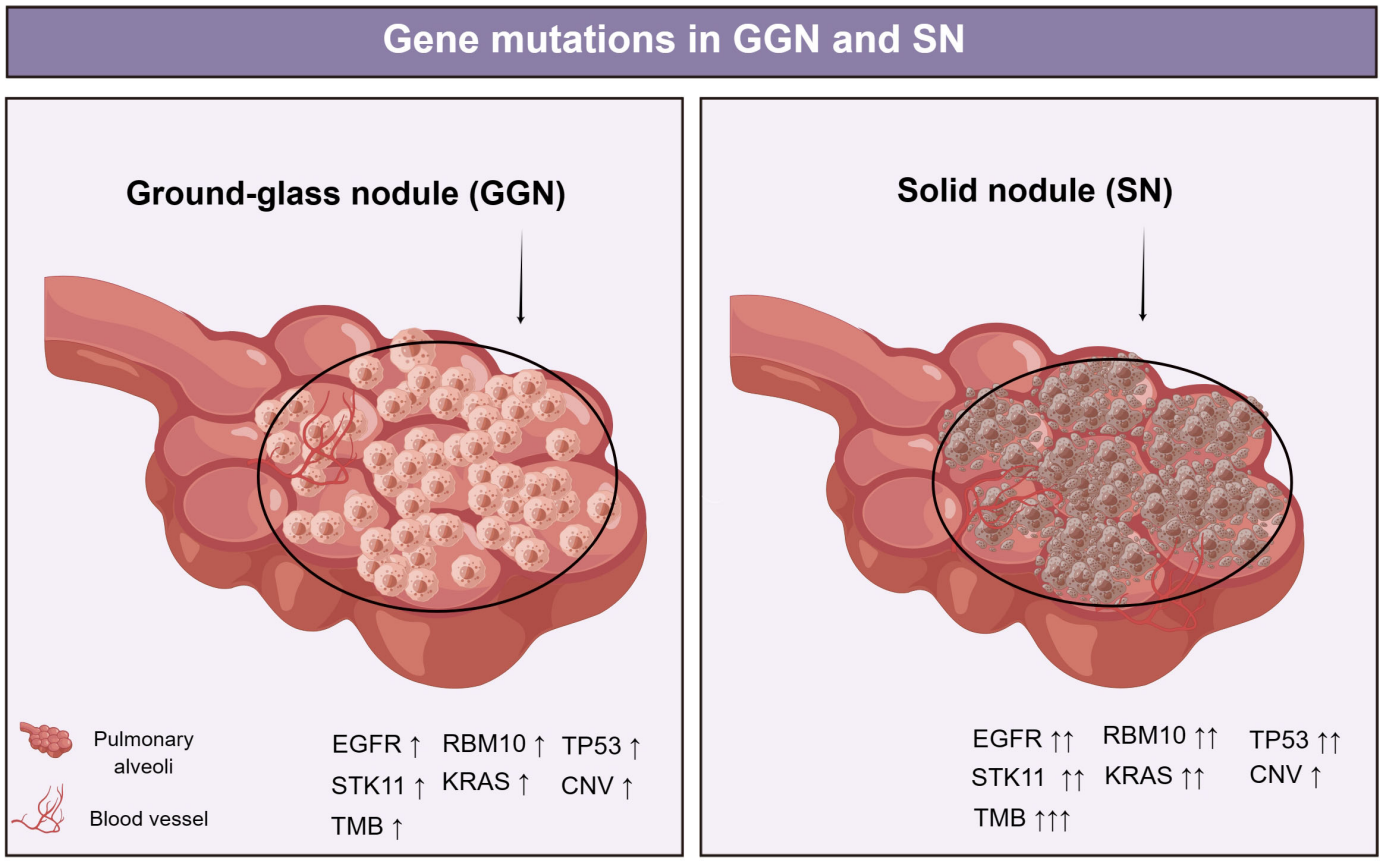
EGFR was the most frequently mutated in GGNs, followed by mutations in RBM10, TP53, STK11, and KRAS. EGFR, TP53, RBM10, and ARID1B mutation frequencies increased with SSNs with a solid component (30). TMB in lung adenocarcinoma (LUAD) presented as SN was significantly higher than that in LUAD with GGO components (35). Li et al. discovered that malignant cells in GGN have CNV patterns similar to those in SN (39). EGFR, epidermal growth factor receptor; RBM10, RNA-binding protein 10; TP53, tumor Protein 53; STK11, serine/threonine kinase 11; KRAS, Kirsten ratsarcoma viral oncogene homolog; CNV, Copy number variation; ARID1B, AT-Rich Interaction Domain 1B.

The tumor mutational burden (TMB) increases gradually from AAH, AIS, and MIA to IAC (13, 30). TMB in lung adenocarcinoma (LUAD) presented as SN was significantly higher than that in LUAD with GGO components, and the proportion of GGO components was negatively correlated with TMB (35). Copy number variations (CNVs) reflect chromosomal instability, and gene gains and losses caused by CNVs can result in tumorigenesis. In a previous study, GGO displayed significantly lower leverage of arm-level CNVs and lower focal CNVs than LUAD, further indicating that GGO had fewer genomic alterations and simpler genomic profiles than LUAD (14). Li et al. discovered that malignant cells in GGNs have CNV patterns similar to those in SN, indicating that different radiological appearances are not correlated with genomic features (39). The CNV patterns of malignant cells within GGNs and solid component cells are similar. This indicates that genomic characteristics do not correspond to radiological characteristics (38). Hu et al. proposed that progression before tumor formation mainly follows the clonal clearance model, whereby partial clonal mutations discovered in early indeterminate lung nodules (IPNs) become clones in later IPNs, whereas inappropriate subclones are eliminated (29). Furthermore, somatic copy number alterations and allelic imbalance may occur during the transition from AAH to AIS and MIA to AIS, respectively (32) (**Figure 1**).

### 3.2 Mutation characteristics of multiple GGN genes

Approximately 20% of patients with GGNs (3% of the screened population) have synchronous multiple ground-glass nodules (SM-GGNs) (11). Clinical decisions regarding the treatment of SM-GGNs are complicated by the difficulty in distinguishing synchronous multiple primary lung cancers from intrapulmonary metastases. According to some studies, SM-GGNs may have a common origin, and intrapulmonary metastasis may occur even in the early stages of lung cancer (11, 31, 40). However, some studies have found that SM-GGNs mostly originate independently (28, 33). There is no genetic linkage between independently cloned SM-GGNs, and they do not have the same driver mutations, nor typical susceptibility mutations. Therefore, they occur randomly (28). SM-GGNs that were not surgically resected did not exhibit changes in size or characteristics during long-term follow-up (41). It is recommended to interpret patients with partially solid GGN lesions as surgical candidates even if they have multiple lesions (42). A good prognosis after surgical resection of SM-GGNs has been reported, supporting the strategy of considering multiple GGNs in patients as primary tumors, and resection may be an appropriate option for the subgroup (33). Most previous studies have shortcomings, such as small sample sizes, relatively low purity of tumors in GGN samples, and geographic limitations of the study population. Thus, in the future, more prospective clinical trials are needed to better understand GGNs in greater depth.

## 4 Immune microenvironment characteristics of GGNs

Targeting tumor cells or the tumor microenvironment (TME) are the two major fundamental principles of antitumor therapies. The TME comprises all the non-cancerous host cells in the tumor and its non-cellular components. One of the most important components of TME is the immune component. During tumor development, a variety of immune cells, including but not limited to macrophages, dendritic cells (DCs), neutrophils, B cells, and T cells, are recruited into the microenvironment surrounding tumor cells, which together with elements such as cancer associated fibroblasts (CAF), and the extracellular matrix constitute the tumor immune microenvironment (43). Accumulating evidence suggests that the tumor immune microenvironment plays an essential role in the regulation of tumor growth and metastasis. Recent findings of studies characterizing the immune microenvironment in ground glass nodules have been summarized in **Table 2**.

**Table 2:** Results of studies reporting characteristics of the immune microenvironment in ground glass nodules.

Author	Publication year	Country	Number of cases	Number of nodules	Image findings	Pathological type	Method	Main conclusions
Lu et al.	2020 (44)	China	10	10	GGN/5, SN/5	NSCLC/10	scRNA-seq	In the GG fraction, CD4+T cells, Tregs, and DC were comparable to those in the solid fraction but significantly higher than in normal lung tissue. Myeloid and NK cells gradually decreased from normal lung tissue and GG fraction to the solid fraction.
Wang et al.	2021 (45)	China	25	25	Pure GGN/25	AAH/3, AIS/5, MIA/9, IAC/17	scRNA−seq, WES	As the pathology progressed from AAH to IA, the exhausted CD8+ T cells and Tregs continuously increased, which inhibited antitumor immunity. The macrophage subtype in precancerous lesions is dominated by alveolar macrophages, whereas that in invasive adenocarcinomas is dominated by tumor macrophages.
Xing et al.	2021 (46)	China	16	16	SSN/16	AIS/1, IAC/15	scRNA−seq	There are more cytotoxic NK cells, T cells, DC cells, and mast cells in SSN than in advanced adenocarcinoma. Epithelial–mesenchymal transition is not found in SSNs, which may account for their rarity of metastasizing. Normal lung tissue and SSN fibroblasts exhibited immunoregulatory features, while mLUAD fibroblasts had tumor-supportive features.
Dejima et al.	2021 (47)	China	53	53	IPN/53	AAH/9, AIS/11, MIA/21, ADC/6	Panel−Seq, WES, TCR−Seq, Methyl−Seq, mIF	With decreasing antitumor immune response from AAH to ADC, immune activation pathways are downregulated, immune suppression pathways are upregulated, cytotoxic T cells and antitumor helper T cells are decreased, and regulatory T cells are increased. Driver gene mutations, chromosomal copy number aberrations, and abnormal DNA methylation may jointly affect the host immune response and promote immune escape.
Chen K, et al.	2021 (35)	China	31	31	(NSN/PSN) 31	AAH/1, LPA/4, MIA/7, ADC/19	RNA-seq, TCR-Seq	GGO-related LUAD has lower expression of immune-related genes, downregulation of immune pathways, less infiltration of immune cells, and lower T cell expansion than Sn-related LUAD. In SN-related LUAD, several metabolic pathways are upregulated, and HLA heterozygosity loss is increased compared to GGO-related LUAD.
He et al.	2021 (15)	China	5	25	Unknown	Normal/12, AIS/3, MIA/5, IAC/5	scRNA-seq	A gradual reduction in T cells and an increasing proportion of macrophages occur as AIS progresses into MIA and IAC. Heterogeneity of tumors increases with the progression of pathology, with CD8 [+] effector T, NK, and myeloid cells facilitating heterogeneity, whereas B cells suppress it.
Li et al.	2022 (39)	China	29	29	GGN/12, PSN/12, nlung/5	ADC/24, Normal/5	scRNA-seq	Solid nodules exhibited significantly higher levels of angiogenesis, epithelial-mesenchymal transition, KRAS, p53, and cell cycle signaling pathways than ground glass nodules. For the tumor microenvironment, the relative abundance of myeloid and NK cells tended to be higher in solid components than in ground-glass components.
Qu et al.	2022 (16)	China	40	50	GGN/25, SN/25	AIS/14, MIA/8, IAC/28	IHC, mIF	Less infiltration of tumor-associated macrophages and T cells was observed in GGO-LUAD. Its TGF-β expression was low, and B and plasma cells were not abundant. PD-1-positive cells were not abundant in GGO-LUAD and SN-LUAD, and there were no significant differences between different imaging manifestations, as well as between different pathological subgroups.
Kim et al.	2022 (48)	South Korea	6	11	GGN/11	IAC/11	scRNA-seq	Fibroblasts associated with tumors negatively regulate protein kinase activity and endothelial proliferation. CD8+ TCS in the early TME and γδ TCS were gradually depleted, while Tregs and B lymphocytes infiltrated more.
Altorki et al.	2022 (17)	USA	62	62	PSN/31, SN/31	AIS/5, MIA/16, IAC/31	WES, RNA-seq	The pre-invasion state is characterized by changes in genes regulating the extracellular matrix and fibroblasts. Various immune cell populations are massively recruited into the microenvironment of pre-invasion/minimally invasive lesions (PSN nodules) and then progress with the development of more invasive malignant tumors.

IPN, indeterminate pulmonary nodule; GGN, ground glass nodule; PSN, part solid nodule; SSN, subsolid nodule; SN, solid nodule; SSN, subsolid nodule; AAH, atypical adenomatous hyperplasia; AIS, adenocarcinoma in situ; MIA, minimally invasive adenocarcinoma; IAC, invasive adenocarcinoma; LPA, adherent adenocarcinoma; mLUAD, LUAD with lymph node metastasis; Panel−Seq, targeted sequencing; WES, whole exome sequencing; TCR−Seq, T cell-infected; body sequencing; Methyl-Seq, methylation sequencing; mIF, multiple immunofluorescent staining; RNA-Seq, transcriptome sequencing; scRNA-seq, single-cell transcriptome sequencing; TME, tumor microenvironment.

### 4.1 T cells

T cells are key mediators of tumor destruction, and their specificity for tumor-expressed antigens is of paramount importance. CD3+ in T lymphocyte subsets is responsible for antigen presentation on the surface of mature T cells and assists in the recognition of T cell antigen receptors. CD4+ T cells are known as helper T cells (Th cells) and act to modulate immune responses by producing cytokines to enhance or suppress inflammation (49). CD4+ T cells can be differentiated into Th1 (antiviral/antitumor), Th2 (allergic), Th17 (autoimmune and pathogen responses), and Tregs (immunosuppressive) (50–53). CD8+ T cells are known as cytotoxic T cells (TC cells) and act to kill pathogen infected or malignant cells by secreting inflammatory cytokines along with cell lytic molecules such as perforin and granzyme (54). Studies have shown that most T cells, macrophages, mast cells, and type I alveolar epithelial cells are derived from normal tissues adjacent to tumors. Most plasma, dendritic, endothelial, and B cells are usually derived from tumor tissues, suggesting that tumors can stimulate their infiltration (15).

In the immune microenvironment, the infiltration of regulatory T (Treg) cells and CD8+ cytotoxic T cells are predominant, whereas the differential Treg genes are enriched in the G2M checkpoint pathways and E2F target pathways (15, 48). In addition, the proportions of effector CD4+ T cells, cytotoxic CD8+ T cells, DCs, and natural killer cells are higher in subsolid nodules (SSNs) than in advanced adenocarcinoma. In contrast, the proportions of regulatory and depleted CD4+ T and depleted CD8+ T are lower in advanced lung adenocarcinoma. These findings suggest that immune surveillance functions well in SSNs (46). CD4+ T cells, Tregs, and DC subtypes in GGN-ADC are comparable to those in solid nodular lung adenocarcinoma (SN-ADC) but significantly higher than those in normal lung tissues. However, the cytotoxicity scores of CD8+ effector memory T cells, gzmk+CD8+ effector T cells, and the natural killer (NK) cell subtypes are reportedly significantly lower in SN-ADC than in GGN-ADC, indicating similar immunity in both radiological components but attenuated cytotoxic function in the more aggressive components (39). This may be because CD8+ T cells derived from GGN-ADC enable cells to function by activating metabolic pathways, oxidative phosphorylation, and enhanced antigen presentation and processing pathways (44). The immunosuppressive microenvironment plays a major role in the occurrence and development of tumors, and T cell-mediated immunosuppression begins in preinvasive nodules and continues to increase in intensity during invasive tumor progression. Increased Treg density and T cell exclusion from cancer nests are the major immunosuppressive mechanisms of pure non-solid (PNS) nodules (17).

T cell types and numbers vary across pathological subtypes. A gradual decrease in CD8+ T lymphocytes, B lymphocytes, NK cells, and granulocytes occurs from normal lungs to AAH, AIS, MIA, and IAC, whereas the CD4+ T lymphocytes and CD4/CD8 ratio increase significantly under such conditions, suggesting that adaptive immune responses and immune escape begin in the preneoplastic stage (15, 32, 45, 47). In addition, exhausted CD8+ T cells and Tregs increase continuously as the pathology progresses, indicating further negative immune regulation as the tumor progresses (47). Meanwhile, as tumor heterogeneity increases with the transition from AIS to IAC, CD8+ effector T cells, NK cells, and myeloid cells act as promoters, and B cells act as suppressors of tumor heterogeneity (15).

T cells are at the core of antitumor immunity in various tumors, and the T cell receptor immune repertoire reflects the strength and diversity of T cell responses; however, chromosomal instability and HLA loss inhibit T cell responses (47, 55). Dejima et al. (47) observed that T-cell clonal strength and clonality were the highest in normal lung tissues and decreased gradually in AAH, AIS, MIA, and invasive ADC. Conversely, T-cell density and diversity increased gradually. They also reported that T-cell receptor oligoclonality and the proportion of the top 10 clonal expansions in PSN-containing GGO components were indistinguishable from those in benign nodules but lower than those in SN, suggesting that T-cell clonal expansion was lower in GGNs (35).In addition, it was found that the infiltration of activated CD4+ T cells, activated CD8+ T cells, and regulatory T cells was negatively correlated with the proportion of GGO components (35).

### 4.2 B cells

B cells play a key role in acquired immunity. They can perform various antibody-independent functions, including engulfing and processing antigens for T cell presentation, secreting soluble mediators (such as interleukin [IL]-10), providing costimulatory signals, and participating in lymphoid tissue development (56). B cells play diverse roles in the tumor immune microenvironment. They can aggregate with T cells and dendritic cells to form tertiary lymphoid structures as an adaptive immune response to persistent tumor antigens (57). Immune escape from tumors is facilitated by regulatory B cells, which have potent immunosuppressive properties (58). GGN-ADC has significantly stronger immune responses of B cells, and antigen processing and presentation are enhanced significantly; NK cell-mediated cytotoxicity and allogeneic rejection pathways are also enhanced significantly in GGN-ADC compared to SN-ADC (44). Studies have shown that B cells in SSN exhibit inflammatory gene expression patterns, whereas B cells in advanced lung ADC are actively transcribed and have a stronger secretory phenotype (46). B cells increase gradually from normal lung tissues to ADC (32). This indicates that as the disease progresses, the number and function of B cells change further.

### 4.3 NK cells

As the primary effector cells of the natural immune system, NK cells play an important role in controlling tumor development (59). NK cells maintain homeostasis through the expression of regulatory receptors related to their killing function. NK cells have two cell subtypes, NK-C1 and NK-C2. Compared to NK-C2, NK-C1 represents the most cytotoxic cluster. Normal lung tissues and SSN have comparable percentages of NK cells; however, NK-C1 cells in SSN have a markedly higher cytotoxic proportion than in LUAD with lymph node metastasis (46). Multiple studies have revealed that NK cell activation induces a stronger immune response in GGN-ADC than in SN-ADC, and the number of NK cells decreases gradually with pathological progression, which may promote a more aggressive PNS nodule nature and immune escape (32, 39, 44).

### 4.4 Macrophages

Macrophages are critical components of the innate immune system. Their functions include phagocytosis, antigen presentation, defense, repair, and metabolism. In GGNs, macrophages tend to polarize toward M1, suggesting a stronger proinflammatory function than in SN (39). Macrophages derived from GGO-LUAD also secrete more proinflammatory cytokines (IL-1 and TNF-α) and chemokines (CCL4), whereas proinflammatory cytokines can recruit cytotoxic T cells to attack cancer cells (44). The macrophage subtype in precancerous lesions is dominated by alveolar macrophages, whereas that in invasive ADC is dominated by tumor-associated macrophages (TAMs), which promote tumor angiogenesis, migration, and invasion and form an immunosuppressive TME (45). In a previous study, TAM exhibited significantly higher infiltration in IAC than in AIS/MIA, whereas TAM infiltration was significantly lower in GGO-LUAD than in SN-LUAD (16). The myeloid subset of AIS is dominated by mast cells, whereas MIA and IAC are dominated by macrophages.

### 4.5 Tumor-associated fibroblasts

CAFs, a heterogeneous population, are the most prominent stromal components outside tumor cells; they can secrete various cytokines to regulate the occurrence and development of tumor cells (60). Fibroblast activation protein-α (FAP) is one of the specific markers of CAFs and is generally not expressed in normal tissue cells but is highly expressed in more than 90% of malignant CAFs derived from epithelial cells; it promotes tumor cell proliferation, metastasis, and immunosuppression (61). A previous study demonstrated that GGN-ADC fibroblasts expressed lower levels of collagen than cells derived from SN-ADC, whereas the cell metabolism of the derived fibroblasts was inhibited (44). However, GGN-ADC also had a similar distribution of fibroblast subtypes as SN-ADC (39). Reproducible detection of myofibroblasts (ACTA2 and RGS5) in AAH and AIS is achievable, indicating that they may represent the TME in early-stage lung ADC (45). Xing et al. discovered that fibroblasts from SSN and normal lung tissues were enriched in immunoregulatory features; however, fibroblasts from advanced lung ADC exhibited tumor-supportive features, such as oxidative phosphorylation, angiogenesis, epithelial–mesenchymal transition, and active transcriptional pathways (46). Furthermore, tumor-related fibroblasts negatively regulate protein kinase activity and proliferation of endothelial cells (48). The preinvasive state is characterized by genetic variations that affect the extracellular matrix and fibroblasts (17).

There are also limitations to the previous research on this topic. First, most of the samples studied were not large enough, and most studies were conducted in Asian non-smoking patients, requiring international collaborative studies in other ethnicities and smokers. Second, the biological complexity of GGNs may vary owing to differences in pathology and single-cell sequencing sampling locations, as well as intra-tumor heterogeneity. In the future, studies of larger cohorts of GGNs are needed to dissect the evolutionary trajectories of GGNs and their underlying molecular mechanisms using tools designed to deal with increasingly complex biological features.

## 5 Other molecular features of GGNs

PD-L1 positivity was observed in AIS, MIA, and IAC, indicating that immunoediting was initiated before invasion (12). Qu et al. observed that PD-L1 expression levels were similar in GGO-LUAD, SN-LUAD, different pathological subtypes, and multiple primary lung ADC (16). However, a meta-analysis revealed that PD-L1 expression in GGO was significantly lower than in SN (34). Furthermore, PD-L1 expression is higher in SN nodules than in PNS nodules, suggesting that immune checkpoint adjustment occurs in aggressive nodules (17). The different findings may be due to significant heterogeneity in the methods and cutoff values used to measure PD-L1 expression. In addition, the expression level of PD-1 differs during the evolution of lung nodules. During the AAH/AIS phase, PD-1 is highly expressed in Pan CK (-) tumor cells; however, in the MIA and IAS phases, PD-1 is expressed highly in Pan CK (+) tumor cells (45). In recent years, the development of immune checkpoint blockade targeting T cells via anti-PD-1 and anti-PD-L1 has revolutionized cancer treatment and has been used to treat early-stage lung cancer and precancerous lesions (62, 63). However, further studies are needed to determine whether treatment in lung nodules with immune checkpoint inhibitors should be based on the level of PD-1 or PD-L1.

Several metabolic pathways were upregulated in SN-LUAD than in GGO-LUAD, indicating that metabolic activity increases with cancer progression (35). The loss of HLA heterozygosity in lung ADC containing GGO components is significantly lower than in those without components containing GGO (35). Hu et al. observed that the proportion of aberrant methylation and intratumoral heterogeneity increased gradually as the pathology progressed; however, increased global hypomethylation was associated with higher mutational burden, CNV, AI, and Treg/CD8 ratio, demonstrating that methylation plays an important role in chromosomal instability and the tumor immune microenvironment during the early development of lung ADC (64). Another study showed that genes in advanced lesions exhibit promoter hypermethylation, which may suppress the expression of neoantigens, leading to antigen depletion and immune escape (47). However, the overall methylation levels were negatively correlated with CD4+ T cell infiltration, CD4/CD8 ratio, Treg infiltration, and Treg/CD8, which further suggests the potential impact of global genomic and methylation statuses on the immune microenvironment (47).

## 6 Treatment of GGNs

As GGNs tend to develop inertly, regular CT monitoring is one of the main international treatment strategies for persistent GGNs (4). GGNs have been studied more and treated more aggressively in Southeast Asian countries (10, 65, 66). And the timing of surgical resection intervention depends mainly on the size of the GGN and the dynamic changes in imaging during follow-up (65). Stereotactic body radiotherapy (SBRT) and radiofrequency ablation are two local therapeutic alternatives, and systemic therapy has been progressively studied in lung cancer with GGNs.

### 6.1 Surgical treatment of GGNs

Video-assisted surgery is increasingly applied to the treatment of GGNs instead of conventional thoracotomy with fewer ports and smaller incisions. Different surgical approaches need to be developed depending on the size and location of the GGNs. Although lobectomy is the standard treatment for early-stage lung cancer because the tumor biology of GGNs may differ from a previous diagnosis of lung cancer, lung segmental resection and lung wedge resection are surgical options. In patients with mixed GGNs, wedge resection should be considered with caution because of the high recurrence rate (67). If the lesion is deeper but still within one of the lung segments, then segmental resection may be considered. If the lesion is located between two or more segments or in a bronchial root segment, then lobectomy is indicated (68). In addition, intraoperative frozen pathology is suggestive of the choice of surgical approach for early-stage lung cancer with GGNs. Multiple studies have drawn similar conclusions and reported no cases of hilar and mediastinal lymph node metastases in GGO-predominant lung tumors, and mediastinal lymph node sampling/selective lymph node dissection is acceptable in patients with c-stage I non-small-cell lung cancer (NSCLC) with GGO-predominant tumors (69, 70).

When multiple GGNs are present in different locations, an appropriate surgical approach based on the principles of surgical oncology that preserves lung function as much as possible is needed (71, 72). Several studies have demonstrated a satisfactory prognosis for patients undergoing resection of multiple GGNs (71–74). Previous studies on the surgical treatment of GGNs have been mostly retrospective, and further large-sample, prospective trials are needed to determine the effect of surgical modality on the prognosis of GGNs as well as interactions between multiple GGNs to determine the optimal surgical treatment strategy.

### 6.2 Nonsurgical treatment of GGNs

SBRT is an alternative therapy for patients with inoperable early-stage NSCLC who are unable or unwilling to undergo surgical resection. Multiple studies have reported no significant difference in survival between patients with stage I lung cancer with GGNs who underwent surgery or SBRT (75, 76). As a precise minimally invasive technique, image-guided thermal ablation has been increasingly used to treat early−stage lung cancer (77–79). Studies have found that either radiofrequency ablation, cryoablation, or microwave ablation (MWA) are safe, minimally invasive, and have good survival rates for the treatment of lung cancer with GGNs (80–82). Huang et al. (83) retrospectively evaluated the MWA efficacy in the treatment of pulmonary multiple concurrent GGNs. The results showed that CT-guided percutaneous MWA for the treatment of multiple simultaneous pulmonary GGNs was feasible, safe, and effective. Radiofrequency ablation may be a suitable option for patients with GGNs who refuse surgery.

Systemic therapy for lung cancer with GGNs has also been reported in some retrospective studies. It was found that chemotherapy was not efficacious against lung adenocarcinoma with GGNs; therefore, chemotherapy should not be a treatment option (84, 85). In multiple primary lung adenocarcinomas, most lesions show multiple GGNs in imaging, many of which have EGFR mutations (86). Cheng et al. (87) conducted a study to determine whether postoperative oral EGFR-TKIs were effective in patients with unresected GGNs with synchronous multiple primary lung cancer (sMPLC) and EGFR mutations. The results showed that EGFR-TKIs showed limited efficacy against these unresected GGO lesions. This may be related to the inability to access the genetic status of each GGN and the heterogeneity among GGNs. Xu et al. (88) showed that the use of immune checkpoint inhibitors was efficacious and safe for patients with sMPLC, which exhibited promise as a neoadjuvant or adjuvant immunotherapy for such patients. Additional multicenter prospective randomized controlled trials are needed to determine whether immunotherapy is effective for treating GGNs.

## 7 Conclusions

Early lung cancer development may be associated with GGO components, and GGO-related molecular features may reflect early lung cancer progression. Clinically, GGNs exhibit indolent development and a favorable prognosis, and multiple studies have reported that GGN-ADC exhibits different molecular characteristics in genomic mutations and immune microenvironments than SN-ADC. We reviewed the prior clinical studies on GGNs alone or including GGNs and outlined the underlying molecular characteristics and immunological features of the indolent biological behavior of GGNs.

Regardless of whether the pathology is AAH, AIS, MIA, or IAC, it can manifest as GGNs on CT. GGNs have different major mutated genes at different developmental stages. In addition, pure GGNs have fewer genomic alterations and a simpler genomic profile and exhibit a progressively evolving genomic mutational profile as pathology progresses. Compared with advanced lung adenocarcinoma, GGN-ADC has a higher immune cell percentage, which is under immune surveillance and has less immune escape. However, as pathology progresses and the solid component increases, negative immune regulation, and immune escape increase gradually, and a suppressive immune environment is established gradually (16, 35, 39, 44, 47, 64).

Some studies have found malignant potential for rapid progression and even metastasis in generally inert GGNs, suggesting heterogeneity in GGNs. Currently, follow-up observation and surgery are the mainstay treatment of GGNs. SBRT and radiofrequency ablation are local therapeutic alternatives, and systemic therapy has been progressively studied in lung cancer with GGNs. Exploration of the characteristics of the molecular evolution of GGNs could provide avenues for distinguishing GGNs with different growth potentials to facilitate the selection of more effective and individualized treatment modalities. In the clinical setting, primary tumor tissue from the same patient is collected only once along the tumor progression trajectory, and serial samples are not available for longitudinal studies. However, with advancements in multiple detection methods, such as imaging omics, circulating tumor DNA, and body fluid metabolomics, it will be possible in the future to reflect the dynamic changes in the GGN genome and immune microenvironment in real-time using multiple detection methods to develop more rational treatment and follow-up protocols.

## Author contributions

FC: Writing – original draft, Writing – review & editing. JL: Writing – original draft. LL: Writing – original draft. LT: Writing – review & editing. GW: Writing – review & editing. XZ: Writing – review & editing.
